# Low-Latency QC-LDPC Encoder Design for 5G NR

**DOI:** 10.3390/s21186266

**Published:** 2021-09-18

**Authors:** Yunke Tian, Yong Bai, Dake Liu

**Affiliations:** State Key Laboratory of Marine Resource Utilization in South China Sea, School of Information and Communication Engineering, Hainan University, Haikou 570228, China; tianyunke19@hainanu.edu.cn (Y.T.); bai@hainanu.edu.cn (Y.B.)

**Keywords:** 5G New Radio, QC-LDPC codes, channel encoding, encoder, low latency

## Abstract

In order to meet the low latency and high throughput requirements of data transmission in 5th generation (5G) New Radio (NR), it is necessary to minimize the low power encoding hardware latency on transmitter and achieve lower base station power consumption within a fixed transmission time interval (TTI). This paper investigates parallel design and implementation of 5G quasi-cyclic low-density parity-check (QC-LDPC) codes encoder. The designed QC-LDPC encoder employs a multi-channel parallel structure to obtain multiple parity check bits and thus reduce encoding latency significantly. The proposed encoder maps high parallelism encoding algorithms to a configurable circuit architecture, achieving flexibility and support for all 5G NR code length and code rate. The experimental results show that under the 800 MHz system frequency, the achieved data throughput ranges from 62 to 257.9 Gbps, and the maximum code length encoding time under base graph 1 (BG1) is only 33.75 ns, which is the critical encoding time of our proposed encoder. Finally, our proposed encoder was synthesized on SMIC 28 nm CMOS technology; the result confirmed the effectiveness and feasibility of our design.

## 1. Introduction

LDPC codes was determined as the 5G NR data channel coding scheme at the 2016 3GPP Conference [1]. After that, the research on implementation of 5G LDPC codes is gradually increasing. In [2], the base matrix of the initial code rate is split, and the smaller sub-base matrix is used to replace the whole base matrix, which improves the efficiency and throughput of encoding and decoding. In [3,4,5], the optimization method of LDPC codes in 5G three scenarios was proposed.

Low latency implementation of LDPC encoding has always been a focus of LDPC application research. For the implementation of the encoder, if the algorithm of multiplying the generator matrix *G* is directly used, the data storage and computational complexity is quadratic in the code length. To address this issue, a simplified algorithm (RU method) is proposed in [6] by transforming the sparse parity check matrix *H* into an approximate lower triangular form to quickly calculate the parity bits. In [7], two encoders based on the RU method have been implemented, but the amount of storage and calculations required increased significantly. After that, through the structural design of the LDPC codes, a quasi-cyclic structure was proposed to greatly reduce the complexity of encoding and the utilization of storage resources.

Some recent studies have focused on the hardware implementation of QC-LDPC codes encoding. Owing to the fact that the encoding complexity of the RU method is lower than that of the direct encoding algorithm, many encoder designs are based on the RU method for structural optimization. The most significant innovation is the parallelized encoding architecture. In [8], an area efficient parallel LDPC encoding scheme is proposed for QC-LDPC codes. This architecture uses multiple parallel cyclic shift network and bit selection algorithm to reduce the hardware complexity. In [9], a multigigabit QC-LDPC encoding architecture is proposed; this architecture leverages the inherent parallelism of QC structural by simultaneously processing multiple bits according to optimal scheduling. In [10], a high-efficiency multi-rate encoder for IEEE 802.16e QC-LDPC codes is proposed; this design uses the double diagonal structure in the parity matrix to avoid the inverse matrix operation that requires a lot of calculations. Meanwhile, a parallel matrix vector multiplication structure and storage compression are used to increase the encoding speed and significantly reduce the number of storage bits required. In [11], a fully parallel QC-LDPC encoder based on a reduced complexity XOR tree designed specifically for the IEEE 802.11n standard was proposed. In [12], a pipeline architecture for QC-LDPC encoder was proposed. The design can be easily reconstructed to support variable code rates and code lengths through parameter configuration. In [13], the encoder stores the matrix vector in random access memory (RAM). The row index of the non-zero entry in each column of the sparse check matrix is used as the write address of the RAM, which reduces the complexity of storage and calculation.

In 5G NR, the channel coding scheme also adopts QC-LDPC codes. For the compatibility of multiple scenarios, the 5G standard has developed two different base graphs, BG1 and BG2, which correspond to two different base matrices, $H_{BG1}$ and $H_{BG2}$. According to the lifting sizes of 5G QC-LDPC codes, the $H_{BG}$ matrix corresponds to a total of 16 parity check matrices (PCM) which defines the 5G LDPC coding schemes [14]. Therefore, the hardware that supports 5G NR codes must provide a high level of flexibility to satisfy different PCMs.

5G NR has three scenarios, enhance Moblie BroadBoand (eMBB), Ultra Reliable Low Latency Communication (URLLC), and massive Machnice Type Communication (mMTC). Specifically, it requires a peak throughput of 10 Gbps for the uplink, 20 Gbps for the downlink, and a user-plane delay of 4 ms for eMBB and 1 ms for URLLC. After evaluation by 3GPP, it is confirmed that the LDPC encoding scheme under BG2 designed for eMBB scenarios is used in URLLC scenarios (mainly low latency) [15]. In [16], a prototype of 5G physical downlink shared channel (PDSCH) transmitter was carried out on software defined radio (SDR), with channel coding experiments including complete processing flow of data transmission in TS38.212, and the system performance of 5G NR was evaluated. In some studies, 5G LDPC encoder is designed according to the complete encoding chain of uplink and downlink channels [17,18], including cyclic redundancy check (CRC) encoding, code block segmentation, LDPC encoding, rate matching, and bit interleaving. By assembling all the processes in the encoding chain, fully functional encoding hardware products can be delivered. At the base station transmitter, channel coding is the crucial operation that affects the bit processing time in physical layer. Therefore, it is necessary to propose a higher parallel encoding algorithm and hardware architecture for 5G QC-LDPC.

There are some references regarding the hardware implementation of 5G LDPC encoder. In [19], an efficient LPDC encoding algorithm was proposed, and a high throughput and low latency encoding architecture is implemented. Synthesis results on TSMC 65-nm CMOS technology with different submatrix sizes were carried out. In [20], a flexible and high-throughput 5G LDPC encoder was implemented on the compute unified device architecture (CUDA) platform through the scheme proposed in [19], and the throughput of 38–62 Gbps from 1/2 to 8/9 rate was achieved on a single GPU. In [21], an encoder with the advantages of parallel encoding and pipeline operation is proposed; it was synthesized in a 65 nm CMOS technology and the parallelism of this scheme is higher compared with [19]. In [22], a serial-optimized QC-LDPC encoder is proposed, which uses genetic algorithm to optimize the encoding. In the case of short codes, multiple check matrix sub-blocks can be partially processed in parallel. The same degree of parallelism as the long code is achieved.

This paper focuses on low latency LDPC encoding specified in 3GPP. One purpose is achieving low power budget in a 5G NR baseband. If we design a LDPC encoder with lower latency, we can give extra computing time in a TTI to other algorithms with heavy power consumption. Thus the degree of parallelization can be relaxed for heavy power algorithms such as channel equalization and detection in uplink [23]. The total power consumption in a base station can therefore be reduced. In order to resolve the issues in the existing schemes, the main contributions of our work are as follows:

(1) The proposed encoder is optimized according to the structure of the parity matrix in 5G standard, which can achieve lower latency and higher throughput than the existing work. Compared with the clock consumption in [19], our design reduces the encoding latency by 56%.

(2) A parallel CRC is seamlessly integrated into the design enabling LDPC encoding from transport block (TB) level, which means that LDPC encoding can be started from TB input. Thus, the complete encoding calculation of PDSCH is implemented.

(3) The encoder is designed to be fully compatible with 5G NR standard and with flexibility and extendibility. Our design uses the largest lifting size as the hardware scale in the shift networks (CNs) of the encoding calculation. Meanwhile, a configurable circuit module is added to CNs to deal with different encoding scenarios. The parameter adaptation module schedules the configurable circuit to change the input and output of the CNs. Hence, the ASIC synthesis of the encoder can support full-size PCM, including all code lengths and code rates encoding for transport blocks (TBs).

Our design is verified and proven by synthesized register transfer level (RTL) design and silicon layout. The IC layout is based on CMOS technology of SMIC 28nm. The results show that at 800 MHz, the encoding time of maximum code length in BG1 is only 33.75 ns (27 clocks), which sufficiently meets the throughput requirement and offers a lower latency for the 5G NR standard.

The rest of this paper is organized as follows. Section 2 describes the coding process of 5G LDPC codes and the structure of the parity check matrix. In Section 3, a high parallel encoding method and the corresponding encoder structure are proposed. The design details and flexible configuration are discussed in Section 4. The silicon verification and comparison results are given in Section 5. Section 6 concludes our paper.

## 2. 5G NR QC-LDPC Encoding

### 2.1. LDPC Encoding Specification in 5G NR

**Figure 1:**
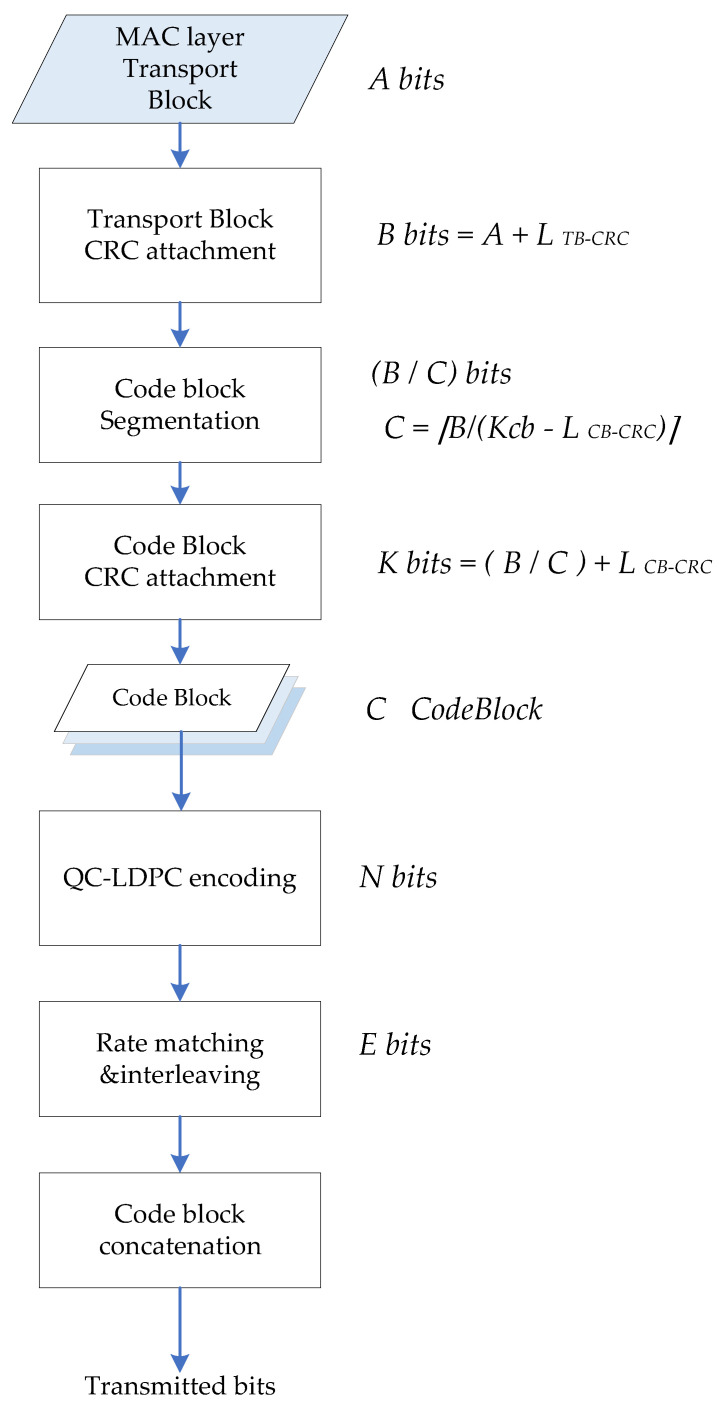
5G PDSCH information transmission process.

In 5G mobile base station, PDSCH channel is used for information transmission at the base station transmitter, and its transmission data is composed of transport blocks (TBs). The information transmission process is shown in Figure 1.

**Figure 2:**
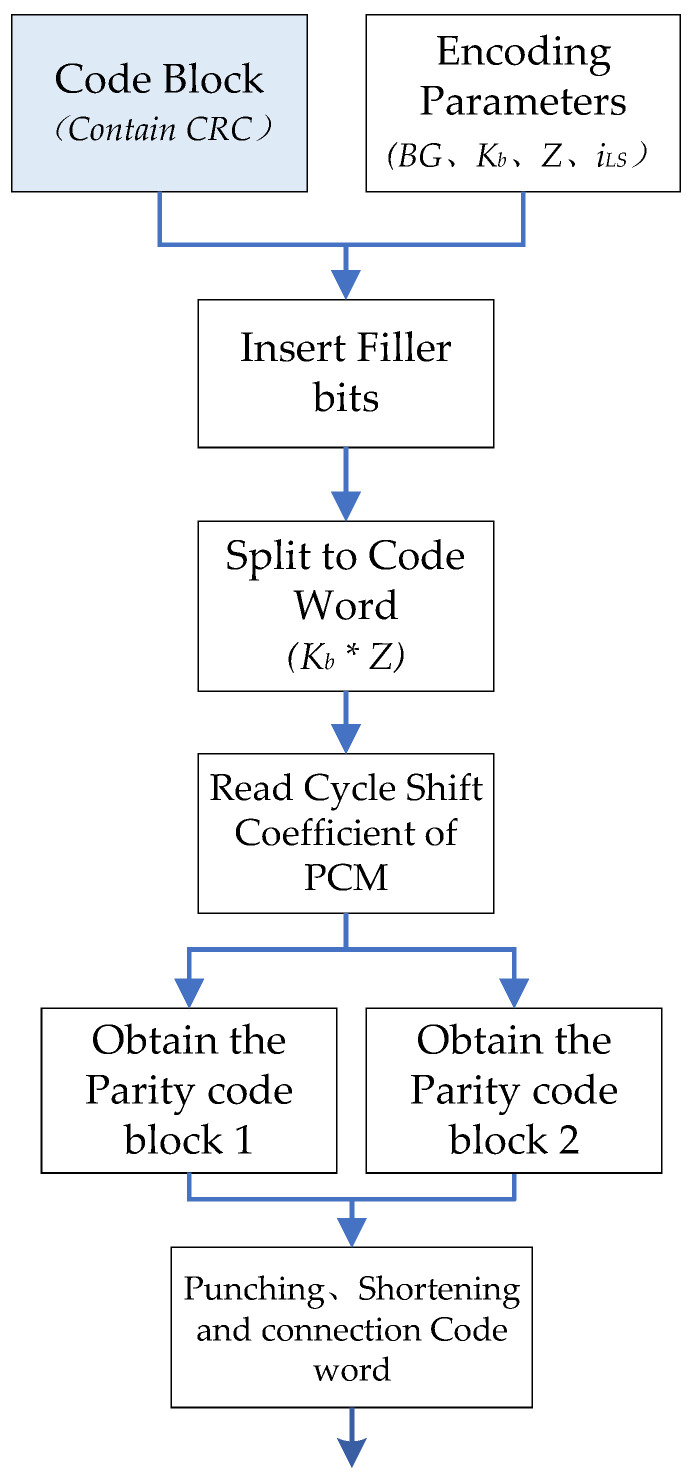
5G QC-LDPC encoding process.

In a TTI, a transmission channel delivers up to two transport blocks to the physical layer, *A* bits TB attaches either 24 bits CRC or 16 bits CRC according to TB length, and TB can be further partitioned to code word (CW). It needs to be divided into *C* code block of *B* bits, each code block will attach 24 bits CB-CRC, and finally it becomes the transport block size (TBS) of *K* bits. $K_{cb}$ is the maximum length of the code block with CRC, $K_{cb}$ is 8448 for BG1, and 3840 for BG2. The base graph and TB length also specifies the number of columns $K_{b}$ in the kernel matrix of the parity check matrix *H*. So $K=K_{b}\times Z$, where *Z* is lifting size and is also the length of each CW. Each *C* code block after segmentation consists of $K_{b}$ CW. After LDPC encoding is completed, a code length of *N* bits is outputted. The encoding output result *N* bits is composed of *n Z*-length CW. The encoding of each *C* code block is independent. After the respective LDPC encoding, rate matching and interleaving are performed respectively. The 5G QC-LDPC encoding process is shown in Figure 2.

### 2.2. Characteristics of 5G QC-LDPC Codes

The matrix *H* is uniquely defined by the matrix $H_{BG}$, extended permutation matrix *P* (also PCM), and lifting size *Z*. The matrix *P* passes through a matrix dispersion, and the elements in *P* are replaced by Z×Z cyclic unit matrix or zero matrix, resulting in a complete parity check matrix *H*. Sets of LDPC lifting size *Z* and its corresponding shift value table are described in the NR standard specification TS 38.212 [1].

**Figure 3:**
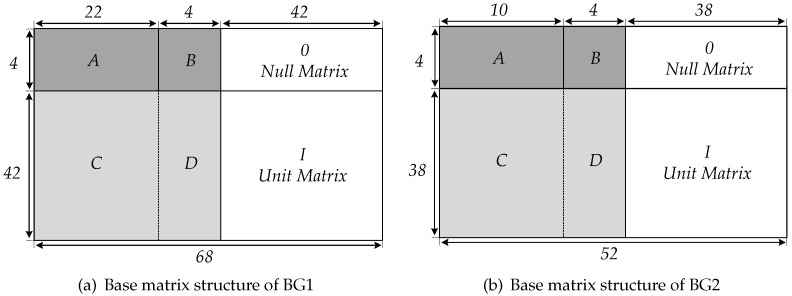
Region division and parameters of base matrix.

**Figure 4:**
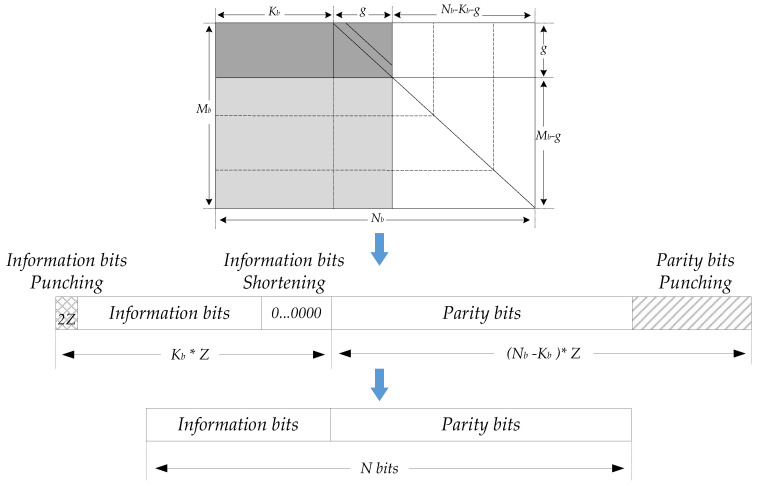
Parity matrix structure and parameters, punching and shortening of 5G LDPC Codes.

Figure 3 shows the parameters and region division of matrix *H* under BG1 and BG2. Region [*A B*] is kernel matrix and [*C D I*] and the all zero matrix in the upper right corner are extended matrix. The kernel matrix can be used to encode information bits at a high bit rate. There are four kinds of *B* matrix corresponding to the parity bits. The *B* matrix adopts a dual-diagonal special structure to avoid complicated operations involved in encoding, such as matrix inversion. When the target code rate is higher than that of the kernel matrix, punching is performed on the parity bits. If the target code rate is lower than that of the kernel matrix, the parity bits with low rate are obtained using the single parity relationship of the extended matrix. Because there are many non-zero elements in the first two columns of the *H* matrix, in order to improve the decoding performance, the information bits corresponding to the first two columns will be punched, Figure 4 shows the matrix structure of 5G LDPC codes and the corresponding punching and shortening operations. 5G QC-LDPC can support any length of code by filling bits at the end of the message and combining with multiple lifting sizes. Through punching operation, it also can support incremental redundancy hybrid automatic repeated request (IR-HARQ) and various code rates.

## 3. Design of 5G QC-LDPC Encoder

### 3.1. QC-LDPC High-Parallel Encoding Algorithm

In [19], an LDPC encoding algorithm for 5G has been proposed. Based on it, this paper optimizes the calculation flow of the parity codeword and arranges the operations in parallel to improve the parallelism of the overall encoding and reduce the latency of the encoder. According to the 3GPP standard, the code block *C* is divided into information code blocks *S*, the first group of parity $P_{1}$, and the second group of parity $P_{2}$, whose lengths are $K_{b}\times Z$, 4×Z, $(M_{b}-4)\times Z$ corresponding to ${\left[A\;C\right]}^{T},{\left[B\;D\right]}^{T},{\left[O\;I\right]}^{T}$ of *H* matrix respectively. $K_{b}$ is the number of columns of the kernel matrix and $M_{b}$ is the number of rows of the *H* matrix, so that $C^{T}$ can be expressed as

(1) $$\begin{array}{c} \begin{array}{c} \begin{array}{c} c^{T}={\left[s\left|p_{1}\right|p_{2}\right]}^{T}={\left[s_{0},s_{1},\cdots ,s_{k_{b}-1}\left|p_{1,1},p_{1,2},p_{1,3},p_{1,4}\right|p_{2,1},p_{2,2},\cdots ,p_{2,m_{b}-4}\right]}^{T} \end{array} \end{array} \end{array}$$

According to the structure of the parity matrix *H* shown in Figure 3, the check equation for encoding is represented as

(2) $$HC^{T}=\left[\begin{array}{ccc} A & B & 0\\ C & D & I \end{array}\right]\left[\begin{array}{c} s^{T}\\ p_{1}^{T}\\ p_{2}^{T} \end{array}\right]=0^{T}$$

The expansion of (2) denoted as

(3) $$As^{T}+Bp_{1}^{T}+0p_{2}^{T}=0^{T}$$

(4) $$Cs^{T}+Dp_{1}^{T}+Ip_{2}^{T}=0^{T}$$

Simplify and obtain $P_{1}$ and $P_{2}$ denoted as

(5) $$p_{1}^{T}=B^{-1}As^{T}$$

(6) $$p_{2}^{T}=Cs^{T}+Dp_{1}^{T}$$

Divide $P_{1}$ into four vectors $P_{11}$, $P_{12}$, $P_{13}$, and $P_{14}$ with length *Z* and assign the result $AS^{T}$ as λ, where $a_{i,j}$ is the element of each row of *A* matrix. $s_{j}$ is the code word of each segment *Z* in the information *S*. This calculation is actually to cyclic shift the Z-length code block corresponding to each element of the PCM, and the number of shifts is the value of the element, that is, the cyclic shift coefficient.

(7) $$\lambda_{i}=\sum_{j=1}^{k_{b}}a_{i,j}s_{j},i=1,2,3,4$$

The encoding operation can thus be divided into two stages: obtaining $P_{1}$ and $P_{2}$. In this paper, the calculation of $AS^{T}$ and $CS^{T}$ is parallelized, and the maximum parallel structures are designed according to the number of columns of *A* matrix and the number of rows of *C* matrix respectively. In the process of obtaining $P_{1}$, in order to avoid the complexity of matrix inversion, traversing each two *B* submatrices of $H_{BG1}$ and $H_{BG2}$, (8–11) shows the four structures of the B matrix, where −1 represents the 0 matrix of Z×Z, 1 and 105 represent the Z×Z unit matrix that is right cyclic shift once and 105 times, and 0 represents the Z×Z unit matrix. Under the principle of GF(2) operations, the process of solving (5) can be converted to (12–15), $P_{i,j}^{\left(\alpha \right)}$ means the right barrel shift of α bits.

(8) $$H_{BG1\_B1}=\left[\begin{array}{cccc} 1 & 0 & -1 & -1\\ 0 & 0 & 0 & -1\\ -1 & -1 & 0 & 0\\ 1 & -1 & -1 & 0 \end{array}\right]$$

(9) $$H_{BG1\_B2}=\left[\begin{array}{cccc} 0 & 0 & -1 & -1\\ 105 & 0 & 0 & -1\\ -1 & -1 & 0 & 0\\ 0 & -1 & -1 & 0 \end{array}\right]$$

(10) $$H_{BG2\_B1}=\left[\begin{array}{cccc} 0 & 0 & -1 & -1\\ -1 & 0 & 0 & -1\\ 1 & -1 & 0 & 0\\ 0 & -1 & -1 & 0 \end{array}\right]$$

(11) $$H_{BG2\_B2}=\left[\begin{array}{cccc} 1 & 0 & -1 & -1\\ -1 & 0 & 0 & -1\\ 0 & -1 & 0 & 0\\ 1 & -1 & -1 & 0 \end{array}\right]$$

(12) $$P_{11}=\left\{\begin{array}{c} \sum_{i=1}^{4}\lambda_{i},whenB=H_{BG1\_B1},H_{BG2\_B2}\\ (\sum_{i=1}^{4}\lambda_{i}{)}^{\left(105modZ\right)},whenB=H_{BG1\_B2}\\ (\sum_{i=1}^{4}\lambda_{i}{)}^{\left(1\right)},whenB=H_{BG2\_B1} \end{array}\right.$$

(13) $$P_{12}=\left\{\begin{array}{c} \lambda_{1}+{P_{11}}^{\left(1\right)},whenB=H_{BG1\_B1},H_{BG2\_B2}\\ \lambda_{1}+P_{11},whenB=H_{BG1\_B2},H_{BG2\_B1} \end{array}\right.$$

(14) $$P_{13}=\left\{\begin{array}{c} \lambda_{2}+P_{12},whenB=H_{BG2\_B1},H_{BG2\_B2}\\ \lambda_{3}+P_{14},whenB=H_{BG1\_B1},H_{BG1\_B2} \end{array}\right.$$

(15) $$P_{14}=\left\{\begin{array}{c} \lambda_{4}+{P_{11}}^{\left(1\right)},whenB=H_{BG1\_B1}\\ \lambda_{4}+P_{11},whenB=H_{BG1\_B2},H_{BG2\_B1}\\ \lambda_{3}+{P_{11}}^{\left(1\right)},whenB=H_{BG2\_B2} \end{array}\right.$$

When $P_{1}$ is obtained, the $CS^{T}$ offers hardware reuse possibility to complete the $DP_{1}^{T}$ operation, and the second group of parity $P_{2}$ is calculated according to (16), where $c_{i,j}$ is the element of each row of *C* matrix, $d_{i,K_{b}+j}$ is the element of each row of *D* matrix, and $s_{j}$ is the code word of each segment *Z* in the information *S*.

(16) $$P_{{{}_{2}}_{i}}\;=\;\sum_{j=1}^{k_{b}}c_{i,j}s_{j}+\sum_{j=1}^{4}d_{i,k_{b}+j}P_{{}_{1j}},i=1,2,\ldots ,m_{b}-4$$

### 3.2. QC-LDPC Encoder Architecture

The encoding algorithm discussed earlier is based on the structural characteristics of the 5G QC-LDPC code; it has the same linear complexity with the RU method. Herein, this paper uses the proposed algorithm for hardware implementation, not only mapping the algorithm to the circuit architecture but also considering the selection and optimization of the circuit architecture in the mapping process and approaching the limit of the LDPC encoding latency.

**Figure 5:**
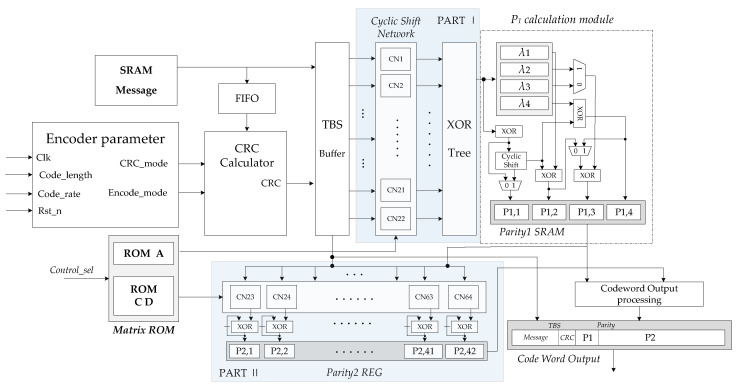
Low latency encoder architecture of 5G QC-LDPC codes.

The overall hardware structure of the encoder is shown in Figure 5. In this encoder, the memory and functional modules are considered as the major influencing factors of the overall area, latency, and power performance of the hardware design. The encoding algorithm used and the operating frequency of the hardware determine the throughput of the overall architecture.

According to the aforementioned high parallel encoding algorithm, the encoding calculation is actually to complete the multiplication of the PCM and the information vector. PCM is composed of Z×Z zero matrix and unit cyclic shift matrix; the multiplication of these submatrices and the information vector is actually a bit-level cyclic shift. Thus, the encoder designed in this paper is implemented by cyclic shift network and combinational logic circuits. The encoding calculation is mainly composed of two parts in parallel, Part I calculates $AS^{T}$ in Equation (5), and Part II calculates $CS^{T}$ and $DP_{1}^{T}$ in Equation (6). In order to realize low latency encoding, 64 (22+42) cyclic shift network (CN) modules are used, which are barrel shift logic. The encoder mainly consists of the following functional modules:

(1) Message buffer SRAM: The transmission information of the media access control (MAC) layer is stored in this module; the information is converted through first input first output (FIFO) to perform multi-byte fast CRC check calculation. Then, the information and its generated CRC bits are transferred to the TBS buffer.

(2) Memory blocks: In Figure 5, the matrix ROM stores the cyclic shift coefficient values of the PCM corresponding to the *A*, *C*, and *D* submatrices.

(3) Encoder parameter calculation module: calculating some parameters required by the encoder according to the code length and the original code rate. Meanwhile, the selected encoding parameters will affect the control signal and state machine of the encoder.

(4) CRC calculator module: executing the CRC calculation of the transmission block with high parallelism.

(5) Transport blocks size buffer: combining information bits and CRC into $K_{b}$ block code words, each code word length is *Z*.

(6) Cyclic shift network Part I and Part II: cyclic shift network is used to implement the cyclic shift of *Z* length code words, according to the cyclic shift coefficient provided by the control signal. CNs is a configurable barrel shift register; corresponding to the *Z* value that does not meet the hardware scale, the input and output of the cyclic shift will be adapted.

(7) $P_{1}$ calculation module: this module consists of combinational logic circuit and memory and configurable circular shift registers. Each $H_{BG}$ has two kinds of *B* submatrices, so this module can flexibly implement different computation processes of different *B* submatrices. Herein, the calculation of Equations (8)–(11) is also parallelized to speed up the process of obtaining $P_{1}$.

(8) Codeword output processing module: punching and shortening the parity code blocks. Connecting the information code block and the parity check code block and output according to the code rate.

According to the column number of matrix *A*, Part I is designed using 22 CN for $K_{b}$ code blocks, which are inputs into each CN sub-module. Meanwhile, one row elements in operations have the current value of CN${}_{1-22}$, equaling to a binary addition operation. The execution in 22 parallel CN sub-modules use four clock cycles to get the intermediate variables $\lambda_{i}\left(i=1,2,3,4\right)$ and to store in memory.

In order to speed up the calculation of (6), Part II may use more CN sub-modules. In each calculation cycle, the same as the execution in each code block of $K_{b}$, a codeword is delivered to CN${}_{23-64}$, and a column elements of *C* matrix is read as the cyclic shift coefficient inputs each CN. When the cyclic shift is completed, the results are kept in registers.

After 22 calculation cycles, 22 column of *C* matrix is used by 42 CN sub-modules. The 42 CN process the cyclic shift of the information code block in parallel. Finally, the outputs of 42 XOR blocks give the last result. After the $CS^{T}$ calculation is completed, the 4 column elements of *D* matrix in ROM are sent into 42 CN in turn, and 4 sets of $P_{1}$ vectors are also delivered to CN${}_{23-64}$ in parallel; a group of results of $DP_{1}^{T}$ are obtained in each cycle. After four times of cyclic shifts and XOR operations, the execution of (12) is completed, and the second group parity $P_{2}$ is obtained. When two groups of parities are obtained, the filler invalid bits are removed by the codeword output processing module. Meanwhile, the first two columns of the information code blocks with *Z*-length are punched out, and the parity codes are punched according to the code rate, then the encoded blocks give output towards the rate matching section.

### 3.3. Execution Pipeline Scheduling

**Figure 6:**
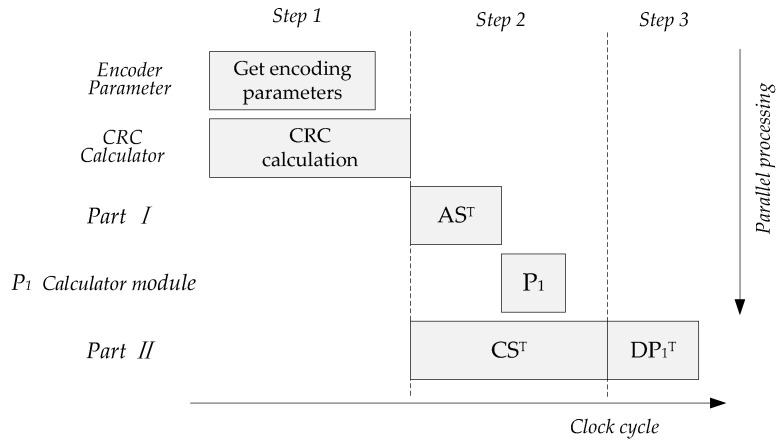
Parallel operation process of encoder.

When the maximum code length is not over the length defined by base graph, the parallel operation process of the encoder is shown in Figure 6, which can be divided into five calculation parts and three steps. The first clock pipeline is for the generation of coding parameters and CRC calculation of TB, the second pipeline is the parallel calculation of $AS^{T}$, $P_{1}$ and $CS^{T}$, and the third pipeline is the calculation of $DP_{1}^{T}$. The latency of the whole encoding process includes CRC calculation and Part II calculation, and the clock cost of LDPC encoding is determined by Part II.

**Figure 7:**
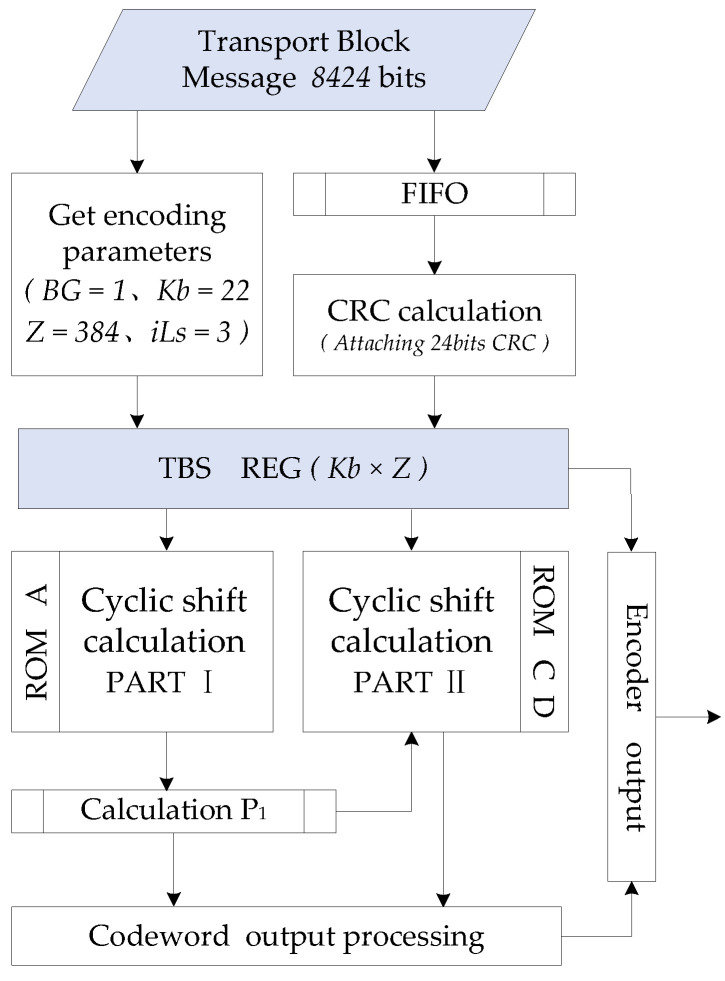
Encoder operation flow chart.

Figure 7 is the flow chart of 8424 bits data encoding operation under BG1. Firstly, the parameters required for encoding are obtained from the transport block data in the encoding parameter module of the encoder. The bit stream is transmitted to the CRC calculation module through the FIFO module, and the CRC is attached to the TB to form the TBS. Then, the elements of the matrix *A* and matrix *C*, *D* are read to complete the encoding calculation in Part I and Part II respectively. After obtaining two groups of parity code blocks, the filler bits of codeword are processed in the codeword output processing module of the encoder; the invalid bits will be removed and the first two columns of information bits and parity bits are punctured. Finally, the information code blocks and the parity check code blocks are connected as the encoder output.

**Figure 8:**
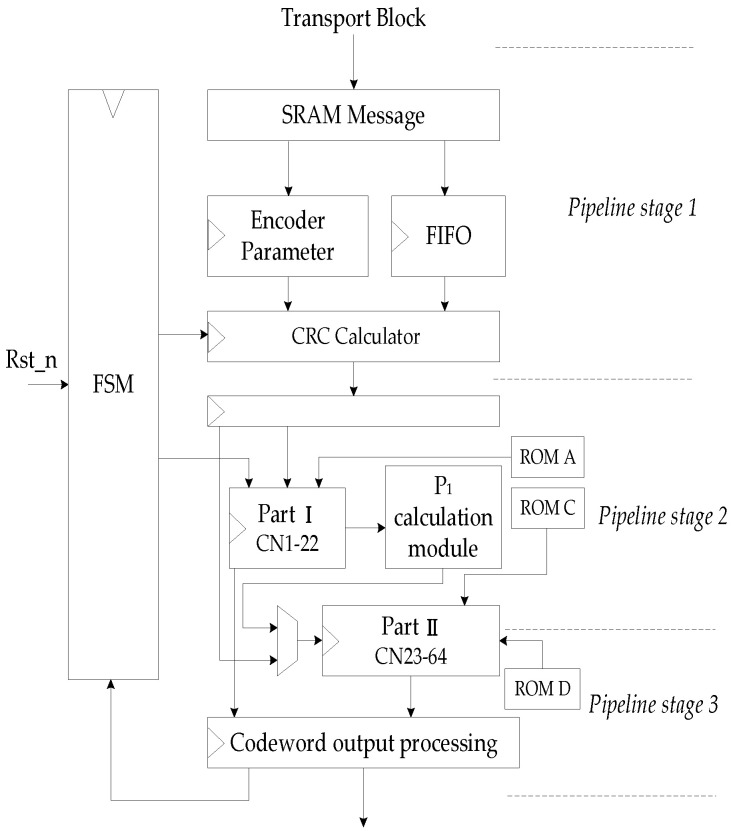
Pipeline of encoder hardware structure.

The control circuit of the encoder issues the overall control of the operation of the encoder, so that each calculation module can orderly perform their own tasks. In this design, the state machine and control signal are used to control the operation of the encoder. It mainly realizes the control signal in sequence to all modules. Figure 8 is the pipeline diagram of the hardware structure of the encoder.

After the parameters are calculated and information bits are stored in SRAM, finite state machine (FSM) issues CRC calculate enable signal. When CRC calculation is completed, the encoder is enabled, the parity check matrix is read in, and the information encoding is carried out. After completing the Part I operation, the calculation of $P_{1}$ starts. After obtaining the first set of parity codes, the hardware waits for the completion of the first round running of Part II, and the input information is switched to $P_{1}$ to calculate the second set of parity codes. When all parity codes are obtained, the encoding end signal is enabled to reset the FSM. The encoder returns to the initial state to perform the encoding of the next frame of information data.

## 4. Encoder Design Details

According to the encoding algorithm and the structure characteristics of the 5G QC-LDPC code, the cyclic shift of encoder and XOR operation in GF(2) is purely logic operations. The hardware complexity of the encoder will increase with the increase of the code length. Especially for CRC calculation, the complexity of encoding hardware will increase linearly with code length. The design of this paper is based on the requirements of 5G NR. In the eMBB scenario, NR requires large-scale data transmission. In the URLLC scenario, NR requires ultra-low latency and high reliability. Hence we need to design an encoder whose performance is close to the latency limit. The solution is to maximize the parallelism of encoding calculations; at the same time, the hardware must be configurable and have reasonable resource utilization. Therefore, this paper optimizes the parallelism of CRC and QC-LDPC encoding and selects a suitable architecture for the flexible configuration.

### 4.1. CRC Implementation in Parallel

The conventional CRC calculation uses LFSR serial calculation method to calculate bit by bit. The processing in serial has low processing efficiency; it is thus almost impossible to complete CRC calculation for large transmission blocks in 5G communication. In this paper, a high-parallel hardware architecture based on look-up table method is designed for CRC calculation.

The CRC calculation module in the encoder was mainly composed of look up table (LUT) structure and XOR gate circuit. LUT is actually composed of SRAM, the CRC value of each byte of data is stored in it. LUT using bytes as the input to avoid the excessive memory resource occupation caused by the increase in the number of input bits. Therefore, the CRC calculation module is designed to decompose the number of bytes of the input bit stream into the number of LUTs.

The look-up table algorithm pre-calculates the specified CRC value for each data byte and stores it in SRAM; through the LUT structure, fast CRC calculation can be performed for long bit stream data [24]. The CRC calculation module divides the input long bit stream into short bits in bytes and uses the LUT to calculate the CRC value of these bytes. Finally, a multi-byte CRC XOR is used to obtain the CRC value of the long bit stream. There are $2^{8}$ situations for bits byte, so that 256 CRC values are stored in LUT; the input of the LUT structure is 8-bits CRC data as the address to obtain the corresponding stored CRC value in the LUT.

**Figure 9:**
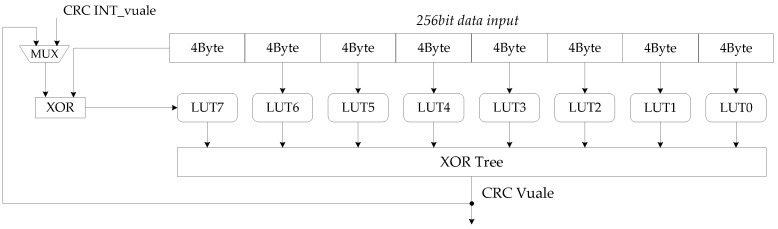
CRC module architecture for 256bits parallel computing.

The maximum code length supported by BG1 is 8448 bits. In URLLC scenarios, BG2 is mainly for short code length encoding. As the communication scenario changes, the amount of data transmitted by the data channel also changes. The length of the TB handed over to the CRC calculation module is less than 8424 on the premise of LDPC encoding. Therefore, this paper designs hardware architecture for processing 256 bits data CRC calculation in one clock, as shown in Figure 9. By implementing low latency CRC computing while maintaining low hardware resource overhead, the overall coding time can be further reduced.

The design of LUTs is in bytes and decomposition of 256 bits is into 32 bytes. A total of 32 LUT units are thus required. To simplify the number of modules in the architecture, we designed a LUT as a module that can look up CRC values for 4 bytes. Therefore, LUT0-LUT7 can perform CRC calculation for 256 bit data. In the process of calculation, there are two problems worthy of attention: when the input data is less than 256 bits, 0 shall be filled in the high significant bits before the CRC calculation; when the input data is larger than 256 bits and cannot be divided by 256, the calculation module will be based on the information of TB length, 0 filled in the high significant bits. Because the CRC value of 0 is also 0, zero-filling will not change the result. The calculation of long bit stream information also starts from the high significant bit.

In each clock, the architecture uses 32 bytes of 256bits data as the index of each LUT look-up table and obtains 32 values. After XOR logic operation, the CRC value (partial remainder) of the 256 bits input data is obtained. The MSB byte of the next 256bits data is XOR with the partial remainder; the result continues to be calculated until all data calculations are completed and the partial remainder becomes the final remainder. The computing latency of 8428 bits inputs is only 33 clocks.The results demonstrate that the CRC calculation module is a suitable design for the overall encoder, and its high parallelism satisfies the fast CRC calculation and maintains a low hardware complexity.

### 4.2. Design for Flexible Configuration

To adapt to variable code length and the eight code rates in the standard, we design a configurable circuit. Code length and code rate as dynamic parameters input into module, configuring static parameters for the following encoding calculations. The encoder parameter module performs parameter calculation via selection and output to the remaining modules after receiving code length and code rate information. The parameters are the base graph BG, the number of kernel matrix columns $K_{b}$, the lifting size *Z*, and the corresponding sets $i_{Ls}$ of *Z*. BG and $K_{b}$ determine the number of iterations for CN. The lifting size reads the memory module for parity check matrix information used in this encoding based on the group to which *Z* belongs. The codeword output processing module punches and shortens the information bits according to the code rate and $K_{b}$, as well as the parity bits.

The encoder hardware scale is designed according to the maximum parity check matrix under BG1, the matrix *H* is constructed by Z×Z size submatrix, and each submatrix is multiplied by the corresponding *Z* length information vector or $P_{1}$ vector. Therefore, the number of CN is set according to the maximum matrix dimension and the fixed length of the barrel shift is set to $Z_{max}$. $Z_{max}$ is used as the CN hardware length to adapt to multiple Z×Z size submatrix. A *Z*-bits as the information or parity is input into CN, if *Z* is less than $Z_{max}$, the LSB vacant bits will be filled with 0 bits, only *Z* significant bits are shifted during cyclic shift, and only *Z* significant bits of each code word are as the output at the end of encoding. Meanwhile, the parity codeword can be achieved according to *Z* as a group. It can also meet the redundancy version requirement of QC-LDPC IR-HARQ transmission.

**Figure 10:**
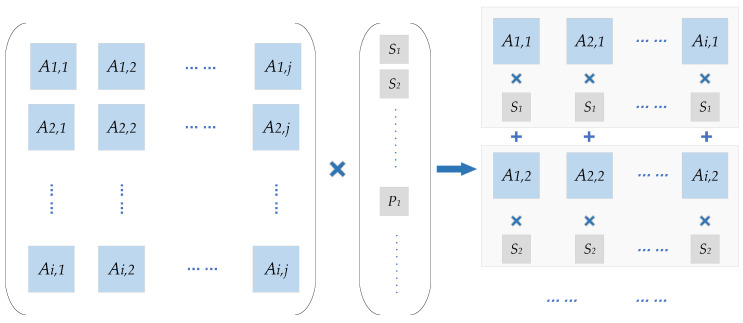
Parallelism improvement of Parities $P_{2}$ calculation.

The encoder hardware shall adapt to changes of code rates. When the code rates are less than 2/5, *C*, *D* matrix has more rows than columns. In order to design a more parallel $P_{2}$ hardware, the multiplication operation between the *H* matrix and the vector is converted as shown in Figure 10. For high code rate, the encoder uses the code word output processing module to punch the corresponding number of *Z*-bits parity codes. With *[C D]* matrix row number as the degree of parallelism, the number of Part II calculation modules is configured. The increase of the CN parallelization degree reduces the encoding delay and improves the throughput.

Therefore, the encoder can achieve code rate compatibility, but the encoding latency of different code rate under different BG depends on the encoding time at the lowest code rate. The proposed encoder needs $K_{b}$ + 4 clock cycles to generate parity sequences under BG1 ($K_{b}$ = 22) and BG2 ($K_{b}$ = 10); it also needs one clock to output the encoded codeword. Therefore, the proposed encoder needs a total of $K_{b}$ and 5 clock cycles to complete the encoding of an information sequence.

The configurable barrel shift register design allows the encoder to be code length configurable. It avoids the waste caused by changing the hardware scale of the encoder for different lifting size. More CN numbers further shorten the encoding latency. The design of the configurable circuit makes the encoder fully meet the requirements of 5G NR flexibility. Although these schemes increase the hardware complexity of the encoder, they have achieved greater improvements in the performance of the encoder such as latency and throughput. Thus it achieves a balance between hardware complexity and encoder performance. We are constantly approaching the limit of the performance of the encoder within the complexity limit that the IC can achieve.

Based on encoding algorithms, we have studied the dynamic configurable scheme of the encoder in this section. We designed a parameterized encoder module; static parameters are available during power on process, and dynamic parameters are changed by L2/L3 setting via MAC-PHY (Port Physical Layer) API (Application Programming Interface). Then, the encoder schedules the hardware to perform encoding operations. The encoder can thus handle variable code length and rate for LDPC encoding through a fixed hardware architecture, which makes the encoder compatible with all configurations following 5G standard.

## 5. Results and Discussions

### 5.1. Discussions and Comparison with Related Work

Presently, there are seldom studies on the hardware implementation of 5G QC-LDPC encoder. In this section, we discuss and compare our proposed scheme with the published state of art to show the differences of related work and explain the novelty of this work.

Reference [19] proposed a 5G QC-LDPC parallel encoding algorithm based on an improved RU method. The encoder uses multiple cyclic shift networks to perform parallel encoding operations. A high throughput and low latency encoding architecture is implemented. Synthesis results on TSMC 65 nm CMOS technology with different submatrix sizes were carried out. The encoding delay is 48 clock cycles, and the throughput ranges from 22.1 to 202.4 Gbps. However, the CRC calculation was not included, and the parallelism of LDPC encoding can be further improved.

Reference [20] adopted the same encoding algorithm as [19] and performs simulation on a single GPU platform. Compared with FPGA and ASIC implementation, GPU-based applications can be flexibly modified and adjusted. The experimental results achieved a maximum throughput of 62.6 Gpbs at the BG2 8/9 code rate. This research verifies the parallelism and throughput of the QC-LDPC encoding algorithm. The implemented encoder is a solution for communication link hardware simulation, which is not applicable to hardware implementation.

For Reference [21], when compared with [19], the maximum encoding latency is reduced by 20 clock cycles. Reference [21] implemented nine encoders for distributed lifting sizes of BG1 and BG2, the application specific integrated circuit (ASIC) synthesis for different lifting sizes. Encoders implemented for different lifting sizes have different performance parameters. Therefore, this work lacks the complete flexibility and configurability for 3GPP standards and cannot meet the needs of all application scenarios.

Reference [22] improved the QC-LDPC encoding algorithm in order to improve the utilization of hardware resources. The proposed architecture is built around the shift network, which improves the original serial encoding structure. The designed flexible shift network has different lifting factors and is divided into three working modes. Partial parallel implementation can provide a compromise between the achievable throughput and the utilization of hardware resources. This paper proposes an optimized coding algorithm and hardware scheduling scheme. Compared with [19,21], the flexibility of the coding architecture has been greatly improved, but the coding delay has not reached the minimum. Under the operating frequency of 580 Mhz, the maximum delay is 875 ns and the peak BG1 throughput is 18.51 Gbps.

In the existing related works, the design of the 5G QC-LDPC encoder is mainly to improve the coding algorithm and design a higher parallel architecture. There are some issues to be solved. There is no design for the complete LDPC encoding chain, such as the design a CRC calculation module. The encoder lacks flexibility and compatibility and the overall architecture cannot satisfy all the lifting sizes, which means that the encoder cannot encode for all PCM.

In the work of this paper, in order to implement a lower latency 5G QC-LDPC encoder, we use more CN modules and increase the calculation speed of the second group of parity check bits (described in Section 4.2). We designed and implemented a higher degree of parallelism encoder architecture. The latency of the proposed encoder is only 33.75 ns at 800 Mhz, compared with the existing work, achieved a significant shortening. Meanwhile, the encoder fully meets the variable code length and code rate in the 5G scenario and improves the compatibility of the CNs to meet all the lifting sizes. Finally, the encoder designed a parallel acceleration structure for CRC calculation, so that the encoder can execute a complete 5G QC-LDPC encoding process.

### 5.2. Evaluation Results

The RTL code of our designed encoder has been validated using Modelsim, synthesized using Synopsys Design Compiler, and placed and routed using Synopsys IC Compiler. Two scenarios are for simulation. Scenario 1 is encoded under BG1. Two information code block lengths are used, $A_{1}$ = 8424 bits and $A_{2}$ = 1920 bits. After attached TB-CRC, and $K_{1}$ = 8448 bits, $K_{2}$ = 1936 bits, the initial code rate is $R_{1}$ = 1/3, $R_{2}$ = 8/9. Scenario 2 information length is 3824 bits, code rate is 1/5, and encoding is under BG2. The system clock is 800 MHz, the total encoding delay of Scenario 1 is 33.75 ns, and the total encoding delay of Scenario 2 is 18.75 ns.

Similar to [25], Table 1 compares the hardware parameters and performance indicators of the proposed encoder implemented by the ASIC with similar references. Throughput in the table is calculated according to Equation (17), where *N* represents the total encoded output that has not undergone codeword processing, *R* is the code rate, $f_{max}$ is the highest frequency of the system clock, and *CC* is the cycle clocks consumed to obtain all parity checks.

(17) $$Throughput=\frac{N\times R\times f_{\max}}{CC}$$

**Figure 11:**
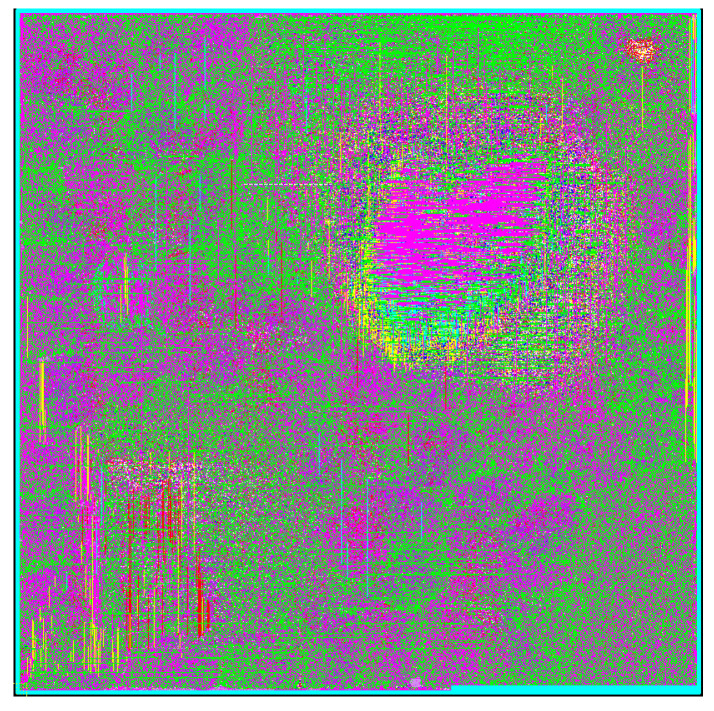
5G NR QC-LDPC encoder layout.

The layout based on the CMOS technology synthesis with SMIC 28nm is shown in Figure 11, because SRAM is replaced by register file; it is a scattered flattening place and routing layout, so the functional module division is not marked in the Figure 11. The silicon layout shows that the total equivalent gates are about 1126K cells, and the total silicon area is 0.712 mm${}^{2}$. The peak dynamic power consumption under 800 Mhz is 123.5 mW. The throughput/area is defined as the normalized throughput area ration (TAR), which is 362 Gpbs/mm${}^{2}$ in this design. The area shows the resource usage of the encoder, and the number of equivalent gates represents the complexity of the implementation. Under BG2, when Z = 384, 3840 information bits are encoded to output 19,200 bits, and the encoder achieves the largest equivalent bit operations per clock: 1280 bit/cycle.

In the proposed architecture, a configurable barrel shift register uses approximately 13.45K equivalent gates; the encoder uses 64 CNs, so CNs and configurable circuits utilize the largest hardware resources. In addition, the LUT and XOR tree structure of 256-bit parallel CRC calculation uses the remaining resources. Although this design is 40% higher than [21] in terms of resource utilization, the proposed encoder is suitable for full-size *Z* and includes CRC calculation. In [19,21], ASIC synthesis is performed for different lifting sizes, and five and nine encoders are implemented respectively. Thus these encoders can only work under the specified *Z* size. Moreover, Refs. [19,20,21,22] do not include CRC calculation. Therefore, the work implemented in this paper has more flexibility for various 5G application scenarios. The entire calculation process of data channel coding in the 3GPP standard has also been implemented. Consequently, the design of this paper is a low-latency hardware structure with complete encoding functions.

From the implementation results, the dynamically configurable architecture designed in this paper can directly perform full *Z*-size encoding operations; it can be seen that this design can be applied to different submatrix sizes. The design has significant silicon area efficiency and encoding throughput, and the design can be applied to a variety of code lengths and code rates.

## 6. Conclusions

In this paper, a 5G QC-LDPC encoder including CRC calculation is proposed. The designed hardware architecture has high parallelism and flexibility. Through parameter configuration, LDPC codes of various code lengths and code rates in the 5G standard can be encoded. This is achieved by improving the cyclic shift structure, the encoder uses the maximum lifting sizes of the 5G standard as the hardware scale and uses encoding parameters to constrain the hardware to achieve effective compatibility of the encoder.

In addition to architecture innovation, the encoder includes the parallelization of CRC calculations and designed a 256-bit parallel CRC calculation architecture based on look-up tables. Therefore, the encoder can perform the complete process of 5G LDPC encoding, which is an innovation compared to existing work.

The hardware implementation of the encoder is based on SIMC 28 nm CMOS technology. Compared with existing similar LDPC encoders, the proposed encoder achieves higher throughput, lower encoding latency, and the equivalent bit operation of each clock is also improved. Hence, our design achieves a better balance among flexibility, coding efficiency, and hardware power consumption and is suitable for 5G eMBB and URLLC scenarios.

## Figures and Tables

**Table 1:** Comparison of hardware implementation of 5G QC-LDPC and other standard LDPC encoders.

Encoder	Standard	Technology	Implemented Codes	$f_{\max}$ (MHz)	CC	Throughput (Gbps)	Area (Resource) (mm${}^{2}$)	Gate Counts (Complexity)	Equivalent Bit Operations Per Clock (Bit/Cycle)
[19]	5G NR	ASIC 65nm	(BG1,Z144)	645	48	89.0	0.171	214K	198
[19]	5G NR	ASIC 65 nm	(BG1,Z352)	600	48	202.4	0.389	486.4K	484
[19]	5G NR	ASIC 65 nm	(BG1,Z96)	714	48	43.8	0.117	146.3K	132
[20]	5G NR	CUDA GPU	(2178,1936)	1770	48	62.6	-	-	41.5
[21]	5G NR	ASIC 65 nm	(BG1,Z384)	575	28	173.7	0.511	639.5K	905
[21]	5G NR	ASIC 65 nm	(BG2,Z352)	586	16	128.9	0.435	545.2K	1100
[26]	GF($2^{2}$)QC	ASIC 28 nm	(2016,1764)	400	256	6.3	0.007	8.66K	7.875
[27]	IRA-LDPC	ASIC 28 nm	(568,512)	-	57	3.57	-	58.5K + (512×64) SRAM	9.96
Proposed ${}^{1}$	5G NR	ASIC 28 nm	(BG1,25344,8448)	800	27	257.9 *	0.712	1126K	938
Proposed ${}^{1}$	5G NR	ASIC 28 nm	(BG1,2178,1936)	800	27	62.0 *	0.712	1126K	80.7
Proposed ${}^{1}$	5G NR	ASIC 28 nm	(BG2,19200,3840)	800	15	213.0 *	0.712	1126K	1280

${}^{1}$ Encoder implementation satisfies full lifting size *Z* and includes CRC calculation. * Based on Equation (17), other throughput is derived from references.

## Data Availability

Not applicable.
